# Efficacy of dual PI-3K and mTOR inhibitors *in vitro* and *in vivo* in acute lymphoblastic leukemia

**DOI:** 10.18632/oncotarget.2260

**Published:** 2014-07-25

**Authors:** Jacky Wong, Robert Welschinger, John Hewson, Kenneth F. Bradstock, Linda J. Bendall

**Affiliations:** ^1^ Centre for Cancer Research, Westmead Millennium Institute, University of Sydney, Westmead, Australia; ^2^ Department of Haematology, Westmead Hospital, Westmead. NSW. Australia

**Keywords:** Acute lymphoblastic leukemia, Cell Signaling, Animal Models, PI-3K, mTOR

## Abstract

The major regulators of human acute lymphoblastic leukemia (ALL) cell growth and survival mediate their effects through the phosphoinositide 3-kinase (PI-3K)/mammalian target of rapamycin (mTOR) pathway. We have shown that the mTOR inhibitor everolimus extended survival in a non-obese diabetic/severe combined immune-deficient (NOD/SCID) mouse xenograft model of human ALL. Since PI-3K has mTOR dependent and independent functions we examined the effect of the dual PI-3K/mTOR inhibitors BEZ235 and BGT226. These agents inhibited the proliferation of ALL cell lines with a three log greater potency than everolimus. However, the induction of cell death differed, with BGT226 being cytotoxic in the low micromolar range while a two log higher concentration of BEZ235 was required to produce the same effect. While all three agents extended the survival of NOD/SCID mice engrafted with human ALL, the responses of individual xenografts varied. Although differential phosphorylation of AKT on Ser^473^ and Thr^308^ in response to everolimus exposure was observed, this did not entirely explain the different *in vivo* responses to the drugs. Our data suggests that while dual PI-3K/mTOR inhibitors may improve therapeutic outcomes for a subset of ALL patients, patient selection will be important, with some patients likely to respond better to single mTOR inhibition.

## INTRODUCTION

Acute lymphoblastic leukemia (ALL) is the most common childhood cancer and a major cause of death in children. Although pediatric ALL is highly responsive to chemotherapy, relapse occurs in approximately 25% of children with ALL [1]. The majority of adults diagnosed with ALL relapse following treatment and their outlook is bleak, with further treatment including hematopoietic stem cell transplantation producing less than 10% overall survival at 5 years [2, 3]. In addition, the relatively non-specific actions of anti-cancer drugs often result in unacceptable toxicities that can occasionally prove fatal, or produce life long consequences for survivors [4]. The inability to further intensify current treatments in high-risk patients due to dose limiting toxicities means that new agents are required for further significant increases in overall survival.

ALL cells are highly dependent on bone marrow stromal support for *in vitro* proliferation and survival [5], and bone marrow stroma can provide protection from the cytotoxic effects of chemotherapeutic agents [6], an effect at least partly mediated by chemokine (C-X-C motif) ligand 12 (CXCL12) [7]. We have demonstrated that signaling through PI-3K/AKT/mTOR is crucial for proliferative responses of ALL cells to CXCL12, interleukin (IL)-7 and unknown stroma-derived mediators [8]. In addition, constitutive activation of the PI-3K/AKT/mTOR pathway has been observed in hematological malignancies including ALL [9], making the PI-3K/mTOR pathway a potential therapeutic target for the treatment of this disease.

We and others have shown that the mTOR inhibitors everolimus, rapamycin, CCI-779 or AZD8055, suppress proliferation, induce cell death and extend survival of NOD/SCID mice engrafted with human ALL [10-13]. However, signaling events elicited by PI-3K and mTOR are complex and although overlapping, have non-identical functions that regulate cell growth and survival [14-18]. Inhibitors of mTOR disrupt mTOR complex 1 (mTORC1), inhibiting phosphorylation of ribosomal protein S6 kinase (S6K) and eukaryotic translation initiation factor 4E binding protein 1 (4E-BP1), while PI-3K signals through a range of other factors that regulate proliferation and survival independent of mTOR [19, 20]. We therefore hypothesized that dual inhibition of PI-3K and mTOR would provide a superior outcome in ALL as compared to inhibition of mTOR alone. Since such inhibitors are entering clinical trial for a range of advanced solid malignancies, including endometrial and breast cancer, if effective, rapid translation of these agents into clinical practice could be anticipated. While a recent study demonstrated superior *in vitro* activity of the dual PI-3K/mTOR inhibitors over mTOR inhibition alone in ALL *in vitro* [21], we extended these findings to the *in vivo* setting using a human ALL xenograft model in NOD/SCID mice. While we confirmed this increased *in vitro* activity of dual inhibitors, this did not fully translate into improved survival times in NOD/SCID mice engrafted with human ALL.

## RESULTS

### The dual PI3K/mTOR inhibitors show greater anti-proliferative effects than mTOR inhibitors in pre-B-ALL cell lines *in vitro*

**Figure 1:**
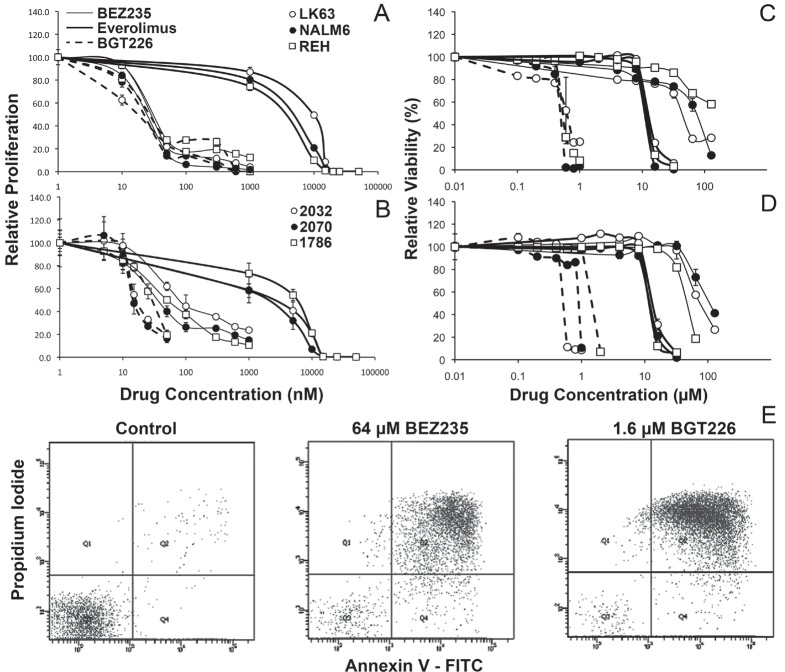
Variable effects of PI-3K/mTOR inhibitors on ALL cell proliferation and survival ALL cell lines (A) or patient derived stromal dependent cell lines (B) were cultured with the indicated concentration of the specified inhibitors for 24h and assessed for proliferation using ^3^H-thymidine. Data was normalized to control values and the mean±SD of quadruplicates are shown. ALL cell lines (C) or patient derived stromal dependent cell lines (D) were cultured with the indicated concentration of the specified inhibitors for 24h and assessed for viability by flow cytometry using annexin V and PI staining. Data was normalized to control values and the mean±SD of duplicates are shown. (E) Representative dot plots showing annexin V/PI staining following exposure to the indicated drug concentrations in NALM6 cells.

We have previously reported that the mTOR inhibitor everolimus reduces ALL cell proliferation and induces cell death [10, 11]. Considering that PI-3K is upstream of mTOR, and is activated by many microenvironmental cues important for ALL proliferation and survival [8, 22], we considered that inhibition of PI-3K in addition to mTOR would be a superior strategy to inhibition of mTOR alone. We compared the effects of the dual PI-3K/mTOR inhibitors BEZ235 and BGT226 with that of the mTOR inhibitor everolimus. BEZ235 and BGT226 inhibited the proliferation of three ALL cell lines (NALM6, REH and LK63) with a similar IC_50_ of between 13 and 26 nM, 2 logs lower that of everolimus (1.5 - 10 μM) (Figure 1A). Using stromal-dependent patient derived cell lines we found BGT226 had similar IC_50_ values of between 13 and 30 nM but BEZ235 was slightly less effective in this setting with IC_50_s of 33, 40 and 80 nM for 2070, 1786 and 2032 cell lines respectively. Despite this the dual PI-3K/mTOR inhibitors were still dramatically more effective than everolimus (IC_50_ 1.5-6 μM) (Figure 1B). Dual PI-3K/mTOR inhibitors are clearly more potent at inhibiting ALL cell proliferation than mTOR inhibitors.

### The dual PI3K/mTOR inhibitors show variable cytotoxic potency and death mechanisms in pre-B-ALL cell lines *in vitro*

The effect of dual kinase inhibitors on ALL cell viability was assessed using annexin V/propidium iodide (PI) staining by flow cytometry and representative dot plots are shown in Figure 1E. In contrast to effects on proliferation, the dual PI-3K/mTOR inhibitors had a more varied effect on cell viability. Over a 24-hour period, BGT226 induced ALL cell death at low micromolar concentrations (IC_50_s of 0.6-1.3 μM) in ALL cell lines (Figure 1C and D and Table 1). In contrast BEZ235 was considerably less potent, requiring between 30 μM and 128 μM concentrations to induce cell death. Everolimus had an intermediate efficacy with IC_50_s of 10 – 13 μM (Figure 1C and D and Table 1) consistent with our previous reports [11]. Extending the assay time revealed increased efficacy of BEZ235 and BGT226 at later time points (p<0.001 for both). IC_50_s for BGT226 fell by 15, 45 and 80% in REH, LK63 and NALM6 cells respectively, and by almost a log for BEZ235 in LK63 and NALM6 over 3 days. This differed from the effect of everolimus, which was not affected by time (Table 1).

**Table 1:** IC_50_ for Viability

Cells	Everolimus (μM)			BEZ235 (μM)			BGT226 (μM)		
Time (h)	24	48	72	24	48	72	24	48	72
LK63	10	10	10	40	3.0	4.0	0.60	0.43	0.33
NALM6	10	10	10	30	15	1.0	1.00	0.83	0.20
REH	11	11	11	>128	46	43	0.60	0.50	0.50
2032	13			73			0.50		
2070	12			110			0.90		
1786	12			46			1.3		

**Figure 2:**
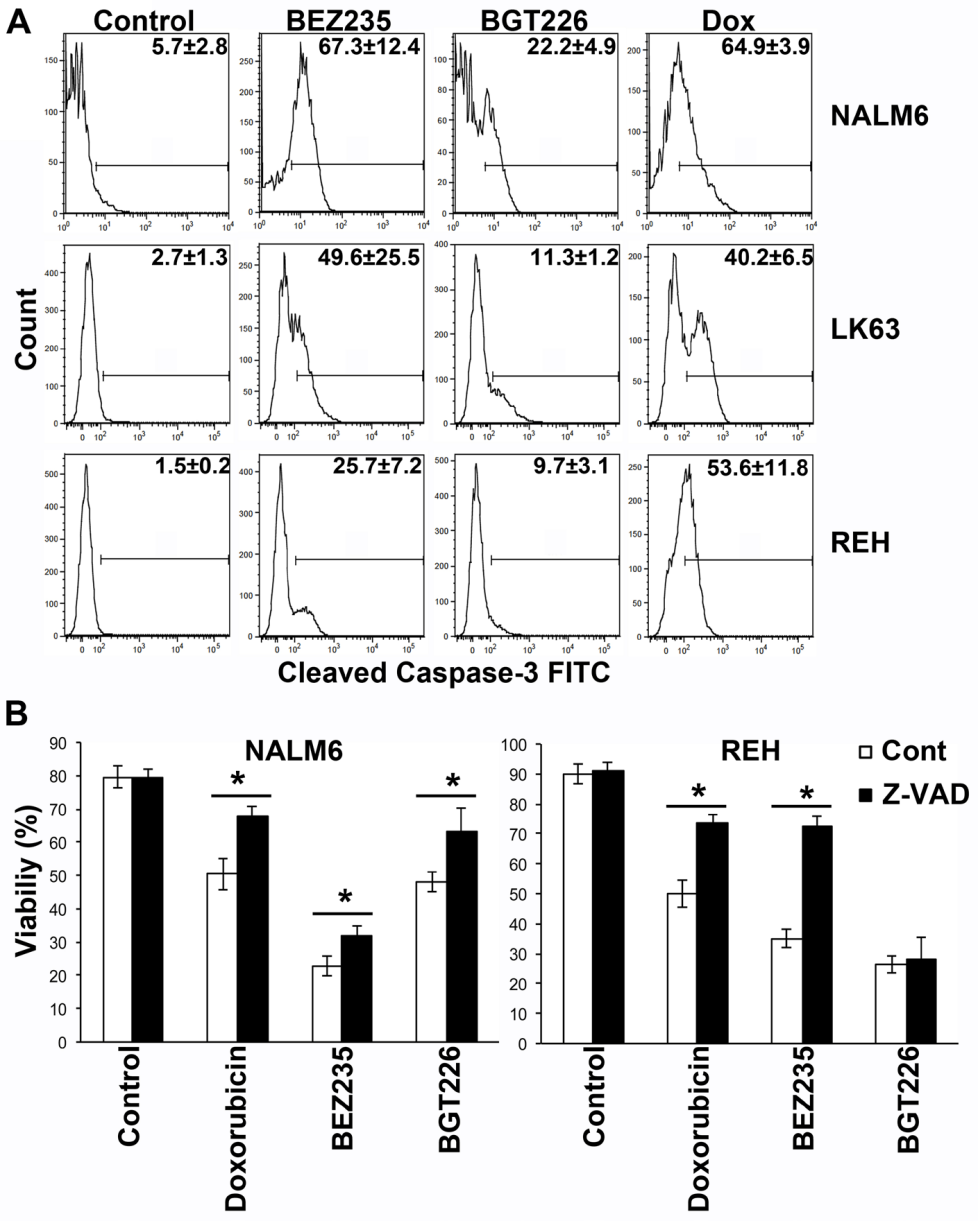
BEZ235 induces caspase-dependent cell death while BGT226 mediates a caspase independent cells death mechanism (A) Cells were exposed to 64 μM BEZ235, 1 μM BGT226 or 0.125 μg/ml doxorubicin for 24 h and caspase-3 cleavage assessed by intracellular flow cytometry. The mean ± SD of the percentage of positive cells from two replicate experiments are shown on each histogram. (B) The indicated cell lines were incubated with 10 mM Z-VAD for 60 min prior to the addition of vehicle alone (Control), Doxorubicin (0.86 μM), BEZ235 (72 μM for NALM6 and 100 μM for REH cells) or BGT226 (0.45 μM for NALM6 and 1 μM for REH cells) and the cells incubated for a further 16 h. Viability was assessed by annexin V/PI staining and mean ± SD of repeat experiments (2 ≤ n ≤ 5). *P<0.05.

**Figure 3:**
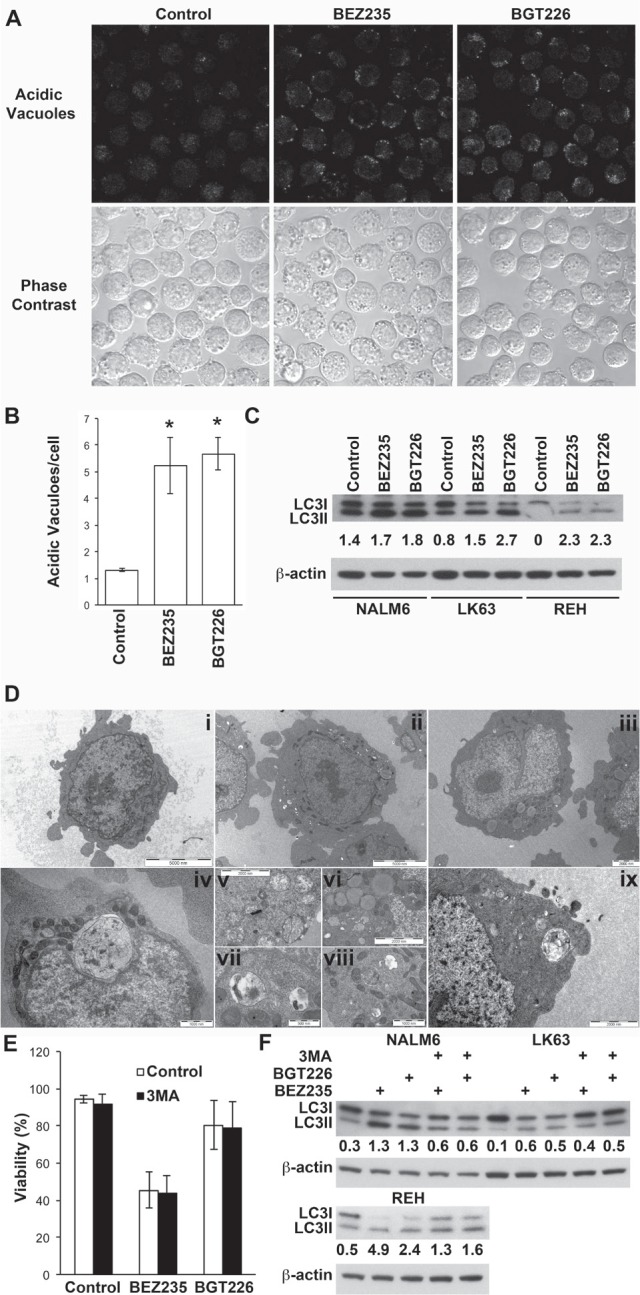
Induction of autophagy by dual PI-3K/mTOR inhibitors (A) NALM6 cells were treated with BEZ235 or BGT226 for the indicated time and stained for acid vacuoles (AV) using lysosensor blue. The upper panel shows lysosensor blue staining while the lower panels phase bright images of the same field of view. (B) Quantitation of AV from figure 4A. The mean ± SE of two experiments is shown. A minimum of 75 cells was assessed for each condition in each experiment. *P<0.05. (C) Western blot of LC3 in ALL cell lines treated with vehicle, or 0.2 μM BEZ235 or BGT226 for 16 h. The ratio of LC3II to LC3I is shown. (D) Electron micrographs of NALM6 cells treated with vehicle (i), BEZ235 (ii, iv, v, vi and vii) or BGT226 (iii, viii and ix). Magnification bars are shown. (E) NALM6 cells were treated with BEZ235 or BGT226 with or without the addition of 5 mM 3MA for 16 h and assessed for viability. The mean±SD of 3 independent experiments is shown. (F) The indicated cell lines were treated as for E and lysates analyzed for LC3.

While BEZ235 induced caspase-3 activation at concentrations associated with the induction of cell death, caspase-3 cleavage was less pronounced following exposure to cytotoxic concentrations of BGT226 (Figure 2A). Consistent with these findings BEZ235-mediated cell death was significantly inhibited by the pan-caspase inhibitor Z-VAD, although the effect was more pronounced in REH than in NALM6 cells. Z-VAD had a similar effect on the viability of BGT226 treated NALM6 cells but was completely ineffective in REH cells where little caspase-3 cleavage had been observed (Figure 2B). This suggests that the death mechanism differs between the 2 agents and that apoptosis is less prominent with the more potent inhibitor. Both inhibitors induced autophagy as evidenced by the development of acid vacuoles (Figure 3A and B), increased LC3 processing (Figure 3C) and the detection of autophagosomes by electron microscopy (Figure 3D), however pre-treatment with 3MA did not significantly affect cell death (Figure 3E) although autophagy was attenuated (Figure 3F).

### BEZ235 and BGT226 show efficacy in a NOD/SCID mouse model of pre-B-ALL but are not always superior to everolimus

In order to examine the potential efficacy of dual kinase inhibitors *in vivo* we used a NOD/SCID mouse xenograft model of human ALL. Mice were engrafted with ALL and treatment commenced when 1% ALL was detected in the peripheral blood. Mice were treated continuously until they succumbed to disease. BGT226 and BEZ235 were used at 40 and 10 mg/kg/daily respectively, the maximum tolerated dose in our model (data not shown). Both BGT226 and BEZ235 increased the overall survival of mice from a median of 37.75 (range 34.5-52, n=6 xenografts with 6 animals/group) days for control treated groups to 71.5 (range 52-105, n=6, p=0.004) days for BEZ235 treated groups and 76.75 (range 67.5-140.5, n=6, p=0.006) days for BGT226 treated groups. As previously reported everolimus treated groups also had an extended survival with a median survival of 78.75 (range 59-97.5, n=6, p=0.003) days. Using a pairwise comparison by performing a Log Rank (Mantel-Cox) test across all 6 xenografts, it was revealed that the dual kinase inhibitors and everolimus resulted in significantly increased survival compared to control, however, the dual kinase inhibitors were not superior to everolimus alone (p=0.23 and 0.36 for BEZ235 and BGT226 respectively), nor were there any overall difference between BEZ235 and BGT226 (p=0.108).

**Figure 4:**
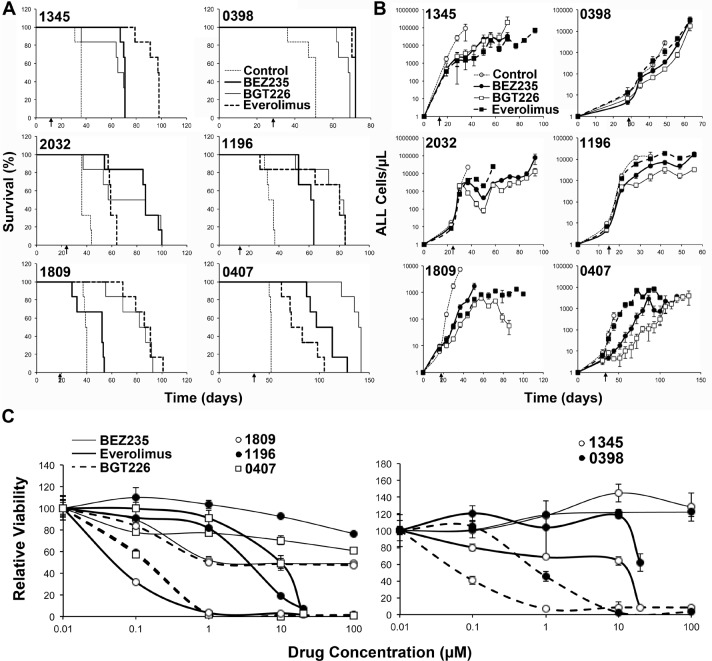
BEZ235 and BGT226 increase survival and reduce ALL in mice NOD/SCID mice engrafted with the indicated human ALL samples were treated as shown when blasts in the peripheral blood reached >1% as indicated by the arrow below the plots. (A) Kaplan-Meier plots of the surviving fraction are shown. (B) The number of ALL cells in the peripheral blood, as determined by weekly bleeds. The start of treatment is indicated by the arrow. (C) The *in vitro* responses of the xenograft cells to the inhibitors as described in Figure 1C.

However when individual xenografts were considered, significant differences between treatments were apparent (Table 2). Each treatment significantly extended survival regardless of the xenograft tested with the exception of BEZ235 in xenograft 1809 (Figure 4). BEZ235 and BGT226 were clearly superior to everolimus in xenografts 0407 and 2032 (p=0.0483 and p=0.0005 respectively for 0407, and p=0.0152 and p=0.003 respectively for 2032). In contrast, everolimus was clearly superior to both BEZ235 and BGT226 in xenograft 1345 (p=0.0012 and p=0.0008), to BGT226 in xenograft 0398 (p=0.0011) and to BEZ235 in xenograft 1809 (p=0.0005). Everolimus also showed significantly improved survival over BEZ235 in xenograft 1196 (p=0.0182). BGT226 tended to perform better than BEZ235, with animals receiving BGT226 surviving longer than those receiving BEZ235 in xenografts 1809 (p=0.0005), 0407 (p=0.0018) and 1196 (p=0.0182), while BEZ235 treated mice survived longer than mice treated with BGT226 in xenograft 0398 (p=0.0011).

**Table 2:** Mean survival time

	0398	1345	1809	1196	0407	2032	Mean
Control	51	36	39.5	34.5	52	36	41.5
BEZ235	72	71	52	62	105	87	74.8
BGT226	68.5	67.5	84.5	81.5	140.5	72	85.8
Everolimus	72	97.5	88.5	80	77.5	59	79.1

Overall xenografts 2032 and 0407 had better responses to the dual inhibitors than to everolimus, while xenograft 1345 had a better response to everolimus than the dual inhibitors. The remaining xenografts had more mixed responses with everolimus performing similarly to one of the dual inhibitors but better than the other. In two of these 3 xenografts BGT226 outperformed BEZ235. To determine whether these differential responses were reflected by differing *in vitro* sensitivities to the agents we repeated the *in vitro* survival studies on the xenograft cells. The order of sensitivity was the same as that observed in the cell lines, with the exception of 1809, which was very sensitive to everolimus *in vitro*, although this was not reflected in the *in vivo* responses of this xenograft (Figure 4C, xenograft 2032 is shown in Figure 1).

### BEZ235 and BGT226 effectively inhibit mTOR signaling *in vivo*

**Figure 5:**
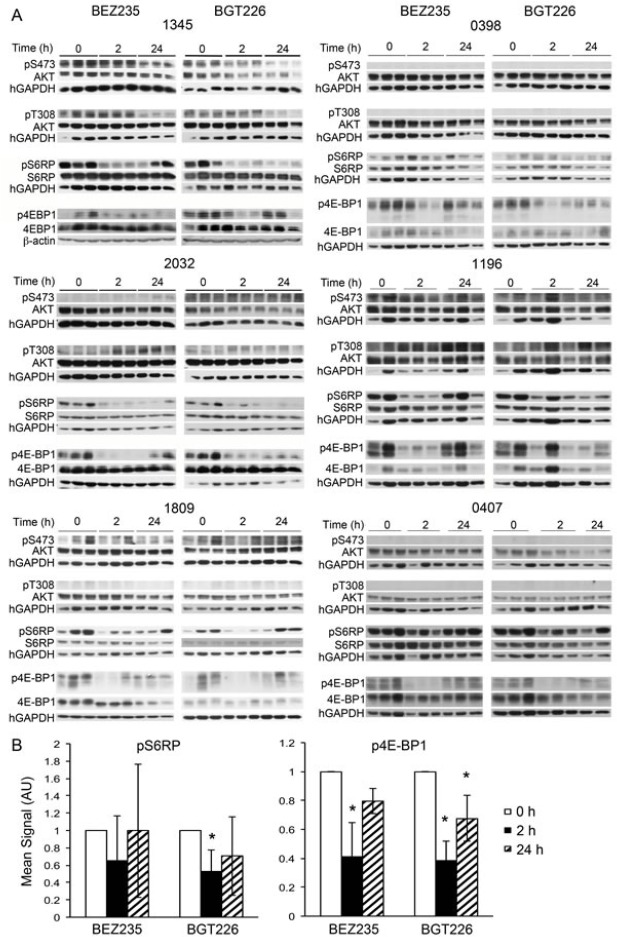
Individual patient samples respond differently to BEZ235, BGT226 and everolimus Western blots of cells recovered from the spleens of mice treated with the BEZ235, BGT226 for the indicated times prior to sacrifice. Treatment was administered when the estimated survival time for untreated animals was less than 2 weeks.

In an attempt to explain the varied responses of the individual xenografts to dual kinase inhibitors we studied the effect of the various inhibitors on signaling events downstream of PI-3K. We have previously reported that everolimus robustly inhibits mTORC1 signaling in our ALL model.[10] Phosphorylation of 4E-BP1 was significantly inhibited by BEZ235 and BGT226 *in vivo* 2 hours after administration (p<0.01 for both agents) but increased by 24 hours, remaining significantly lower than controls for BGT226 only (p=0.02, Figure 5B). Phosphorylation of ribosomal protein S6 (S6RP) was consistently inhibited by BGT226 at 2 hours (p=0.01, Figure 5B) but mixed responses to BEZ235 were seen (Figure S1). By 24 hours, neither agent had an overall significant effect on the phosphorylation of S6RP with only xenograft 2032 remaining significantly inhibited by both agents. Overall, there was no clear association between the extent of inhibition of mTOR signaling and the response of the xenograft to treatment with the dual inhibitors (Figure 4 and 5). Where phosphorylation of AKT on Ser^473^ could be detected it was reduced by BEZ235 and BGT226 at 2 hours and somewhat less so at 24 hours. In the case of Thr^308^ the effect was generally less marked but highly variable between xenografts (Figure 5A).

### Effect of AKT activation on responses to dual kinase inhibition

**Figure 6:**
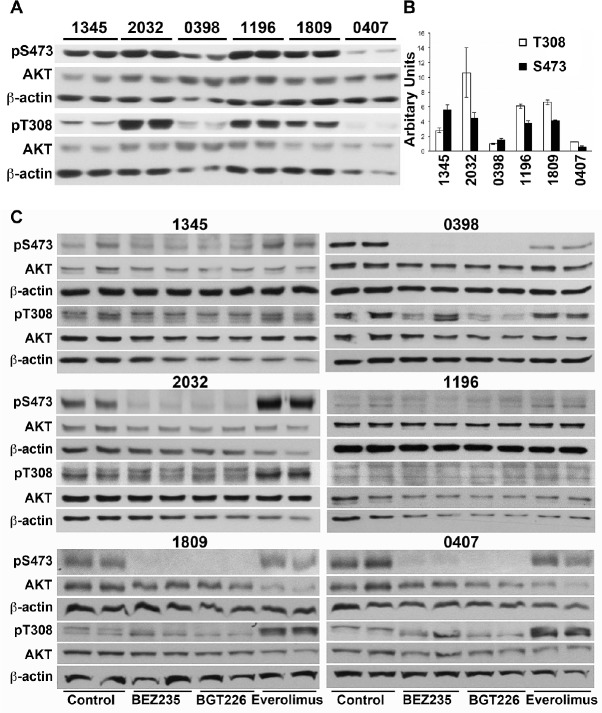
Phosphorylation of AKT in does not predict response to everolimus or dual kinase inhibitors (A) Cell lysates were prepared from xenograft cells recovered from untreated mice and subjected to Western analysis. Lysates from two mice bearing each xenograft are shown. (B) Bands were quantified by densitometry and the mean ± SE of the ratio of phosphorylated to total AKT is shown. (C) Xenograft cells were cultured for 6 hours in the presence of 2 μM everolimus, 0.2 μM BEZ235 or 0.2 μM BGT226 and cell lysates prepared. Lysates were sequentially probed for phosphorylated and total AKT, and beta-actin.

We compared the basal phosphorylation of AKT on Ser^473^, reportedly mediated by mTORC2, and the 3-phosphoinositide-dependent protein kinase 1 (PDK1) specific site Thr^308^. Xenograft 0398 displayed 4-5 more total AKT than the other xenografts (Figure S2A) but the proportion of AKT that was phosphorylated on either The^308^ or Ser^473^ was lower than for other xenografts with the exception of xenograft 0407, which also had low AKT phosphorylation (Figure 6B). Although high phosphorylation of AKT on Ser^473^ was associated with a greater extension of survival by everolimus this did not reach statistical significance (Figure 6A & B). If phosphorylation of AKT was normalized to actin rather than total AKT then 0398 had comparable levels as the remaining xenografts but xenograft 0407 remained lower than the others (Figure S2B). Again there was no correlation between phosphorylation of AKT on either site and the survival of mice treated with any of the compounds.

The effect of the inhibitors on the phosphorylation of AKT on Ser^473^ and Thr^308^ was also examined in *in vitro* cultures. As expected, everolimus did not inhibit but increased phosphorylation on Ser^437^ in four of the six xenografts (2032, 0407, 1809 and 1196) (densitometry shown in Figure S3), presumably due to activation of mTORC2 resulting from inhibition of S6K. Furthermore, everolimus also increased phosphorylation of AKT on Thr^308^ in xenografts 2032, 0407 and 1809, two of which responded better to the dual inhibitors. Increased phosphorylation of AKT was not observed when the dual kinase inhibitors were used. BEZ235 and BGT226 both inhibited phosphorylation of AKT on Ser^473^ in xenografts 0398, 1809 and 0407 but neither agent inhibited phosphorylation on Thr^308^ with the exception of BGT226 in xenograft 0398 (Figure 6C and S2). There was no association between the feedback phosphorylation of AKT on Thr^308^ or Ser^473^ following everolimus and responses to drugs *in vivo*.

## DISCUSSION

The PI3K/AKT/mTOR pathway is aberrantly activated in many cancers, including hematological malignancies [23, 24]. This may result from overexpression of any of the catalytic subunits of class I PI3K or gain-of-function mutations in p110alpha [25], although these mutations are not common in leukemias [26]. Loss of expression and/or function of phosphatases, most notably PTEN, that regulate this pathway have also been reported, including T-ALL [27, 28]. We, and others, have demonstrated that inhibition of signaling through mTORC1 kills ALL cells [11, 29, 30]. The rapalogue everolimus induced a caspase-independent cell death *in vitro* and prolonged the survival of immune-compromised mice engrafted with human ALL [10, 11]. Although everolimus extended recipient mouse survival for each xenograft tested, in some instances the effects were relatively modest and in all but one xenograft the mice ultimately died of ALL.

Signaling through PI-3K can promote cell survival and proliferation through mTORC1 independent mechanisms [14-18]. Furthermore, inhibition of mTORC1 can result in phosphorylation of AKT on Ser^473^ by mTORC2 [31] or on Thr^308^ due to loss of the negative feedback loop through S6K and further activation of PI-3K [32]. Therefore, we considered that dual mTOR/PI-3K inhibitors might provide a superior effect to mTORC1 inhibition alone. In this study we compared the efficacy of two dual specificity ATP-competitive PI-3K/mTOR inhibitors, BEZ235 and BGT226 with the rapalogue everolimus, which is a rapamycin analogue that specifically inhibits mTORC1 [33]. In addition to mTORC1, BEZ235 and BGT226 inhibit all isoforms of PI-3K and mTORC2 [34, 35]. As a result of their additional targets, BEZ235 and BGT226 can block mTORC1 independent PI-3K functions and prevent the two major feedback loops that potentially undermine the efficacy of mTORC1 inhibition. Indeed BEZ235 and BGT226 did not produce feedback activation of AKT and as anticipated BEZ235 and BGT226 had a significantly greater cytostatic effect on ALL cells than everolimus. However, only BGT226 had greater cytotoxic effects, with BEZ235 being less cytotoxic *in vitro* than everolimus. The lower cytotoxicity of BEZ235 as compared to BGT226 identified here is consistent with findings in ALL cells by Badura et al [21]. There was no evidence to suggest that efficacy of any of the agents was dependent on the underlying genetic drivers of the disease as the samples tested had a range of genetic lesions (Table S1) but were equally affected *in vitro*. We have previously shown that everolimus kills cells in a caspase-independent manner [11] but BEZ235 consistently induced caspase-dependent apoptosis as reported by others [36]. The more potent BGT226 had a mixed effect with apoptosis being induced in some cell lines but was not detected in others.

Consistent with superior *in vitro* cytotoxic effects, BGT226 outperformed BEZ235 in three of the six xenografts tested. Surprisingly, neither BEZ235 nor BGT226 proved to be superior to everolimus in extending the survival of xenografted animals when all the xenografts were analyzed. However, it was clear that some xenografts responded better to the dual PI-3K/mTOR inhibitors while others had superior responses to everolimus. We considered that the xenografts that had better responses to dual kinase inhibition might have had higher basal AKT activation or stronger feedback AKT phosphorylation in response to mTORC1 inhibition. However, these factors did not provided a mechanism to identify the xenografts would respond better to dual kinase inhibition. Further exploration of the underlying biology of the xenografts may provide answers. Genome-wide sequencing and gene expression analysis may provide insights into which pathways are driving the individual leukemias, thereby providing insights into likely responses to agents. In addition, the assessment of effect of drug treatment on gene expression and the phosphoproteome of samples may provide insights. However, it is possible that multiple factors/features contribute to the response making it difficult to prospectively identify patients that will respond better to dual kinase inhibitors and those for whom everolimus will produce a superior outcome.

The somewhat disappointing *in vivo* response to dual kinase inhibitors such as BEZ235 has important implications regarding to translation of these agents into clinical practice. The current clinical trial of the rapalogue, everolimus in combination with chemotherapy for adult ALL (NCT00968253) is currently providing promising results [37]. However, the hoped for improvement using dual kinase inhibitors now appears less certain. The lack of any sustained responses suggests that these inhibitors will not be useful as single agents, even in minimal residual disease setting, as resistance is likely to develop. Nevertheless, the dual kinase inhibitors have yet to be tested in combination with standard chemotherapy agents in leukemias, and it is possible that such combinations may provide superior results. The potential for synergistic interactions with DNA damaging agents is suggested by the ability of BEZ235 to inhibit the PI-3K related enzymes DNA-dependent protein kinase (DNA-PK) [38], ATM [39] and ATR [40] that are important for responses to DNA damage [41]. Indeed the recent study by Shortt et al demonstrated that inhibition of these enzymes, along with mTORC1, was responsible for the induction of apoptosis in lymphoma cells [36]. In solid tumors BEZ235 enhanced the effects of cisplatin in nasopharyngeal carcinoma [42] and a range of cytotoxic agents in hepatocellular carcinoma [43]. Whether BGT226 also inhibits these enzymes is current not known and may provide an explanation for the differing cell death mechanisms observed with the two agents, but BGT226 and BEZ235 similarly sensitized endothelial cells to ionizing radiation [44].

Overall the strong responses to everolimus and limited improvement in the presence of dual kinase inhibition suggests that in B lineage ALL signaling through mTORC1 may be the most important arm of the PI-3K/AKT/mTOR pathway in mediating cell survival and proliferation. Indeed the prevention of AKT activation by the dual kinase inhibitors did not necessarily result in *in vitro* cell killing or extended survival times in leukemia bearing mice. Levy et al demonstrated activity of the selective AKT inhibitor GSK690693 in a proportion of ALL cell lines [45], but the failure to affect all cell lines, including those of the B lineage, suggest that AKT is not essential for ALL cell proliferation and survival.

Overall our study shows superior activity of dual PI3K/mTOR inhibitors in suppressing *in vitro* ALL cell proliferation than single mTORC1 inhibition by everolimus. Perhaps surprisingly only BEZ235 consistently induced apoptosis, although this was only achieved at very high concentrations. These effects were consistent across all leukemic cell lines tested, regardless of the underlying genetic changes. While the dual kinases inhibitors significantly extended the survival of ALL bearing mice these effects were variable between xenografts and were not always superior to those achieved with everolimus. The reason for the variable responses is not clear but is not explained by higher basal or feedback induced AKT activation.

## MATERIALS AND METHODS

### Cells

Human precursor-B ALL cell lines were obtained as follows: NALM-6 from Deutsche Sammlung Von Mikroorganismen und Zellkulturen Gmbh (DSMZ, Braunschweig, Germany); Reh from American Type Culture Collection ATCC (Manassas, VA, USA); and LK-63 was a gift from Professor Andrew Boyd (Queensland Institute of Medical Research, Brisbane, QLD, Australia). Cells were maintained in RPMI medium containing 10% fetal calf serum (FCS) (complete media) as previously described [46]. Stromal dependent ALL cell lines were developed in this laboratory by culturing patient samples on stromal layers as previously described [47]. Patient-derived xenografts have been previously reported [10, 48, 49]. A summary of the clinical information relating to the in house cell lines and xenografts has been provided in Table S1 for ease of reference.

### Antibodies and Reagents

Everolimus, BGT226 AND BEZ235 were kindly provided by Novartis (Basel, Switzerland). Vincristine sulfate was purchased from Millennium Pharmaceuticals (Cambridge, MA), Doxorubicin from Pfizer (Melrose Park, NSW, Australia). Ionizing radiation was delivered using an X-ray irradiator (XRAD320, Precision X-Ray, Inc. East Haven, CT) at a dose rate of 0.91 Gy/minute. The following antibodies were purchased from Cell Signaling Technologies (Danvers, MA, USA): rabbit anti-phospho-4E-BP1, rabbit anti-4E-BP1, rabbit anti-phospho-S6RP, mouse anti-S6RP, rabbit anti-phospho-AKT (Ser^475^), rabbit anti-phospho-AKT (Thr^308^), rabbit anti-mouse AKT and rabbit anti-LC3B. Rabbit anti-cleaved caspase 3 was purchased from BD Pharmingen, (San Diego, CA, USA).

### Proliferation Assays

Proliferation assays were performed by ^3^H-thymidine (Perkin Elmer, Glen Waverley VIC Australia) incorporation as previously described [7]. Briefly, ^3^H-thymidine (37 000 Bq) was added after the indicated period of culture and cells harvested onto glass fiber filters (Perkin Elmer) after another 16 h of culture. Radioactivity was measured using a Topcount NXT scintillation counter after addition of 30 μL of Microscint 40 per well.

### Flow Cytometry

Intracellular staining was performed on cells fixed in ice cold 70% ethanol and blocked in perm/wash buffer (BD Biosciences, 554722) containing 10% human AB serum for 1 h. Cells were labeled for 1 h with appropriate primary antibodies, or isotype control antibody, at room temperature in the dark. Cells were subsequently washed and resuspended in 100 μL PBS for analysis by flow cytometry. To assess viability cells were labeled with annexin V (Becton Dickinson, Franklin Lakes, NJ, USA) and propidium iodide (Sigma-Aldrich, St Louis, MO, USA) according to the manufacturer's instructions and analyzed using a FACSCanto flow cytometer.

### Immunofluorescence Microscopy

Cells were treated as described and labeled with 10 μM Lysosensor Blue DND-167 (Molecular Probes, Eugene, OR) as previously described [10]. Cells were resuspended in fresh medium prior to examination using an Olympus FV1000 confocal laser scanning microscope system, based on an Olympus IX-81 ZDC microscope, with BP 330-385 nm excitation and BA 420nm emission filters. Images were captured using FV10-ASW 1.7 software and the number of acidic vacuoles in cells quantitated using ImageJ software.

### Western blotting

A single cell suspension was obtained from spleens and red cells lysed with 0.155 M NH_4_Cl, 10 mM KHCO_3_ and 0.1 mM EDTA (pH 7.5). Cell lysates were prepared and equal amounts of protein loaded in each lane of 7.5 or 15% SDS-PAGE gels and transferred onto nitrocellulose membranes as previously described [22]. Phosphorylated and total proteins were detected sequentially on the same membrane using specific primary antibodies, appropriate secondary antibodies conjugated to HRP and enhanced chemiluminescence (Perkin Elmer, Boston, MA). Bands were quantitated by densitometry (Molecular Dynamics) using ImageQuant software.

### Mouse models

NOD/SCID mice were housed in sterile micro-isolator cages in ventilated racks. Protocols were approved by the Westmead Animal Ethics Committee. Everolimus was formulated at 2% (w/v) in a microemulsion vehicle, BEZ235 at 80 mg/mL and BGT226 at 20 mg/mL (Novartis Pharma AG). 100 μl of freshly thawed drugs were administered by gavage. Everolimus (5 mg/kg) was given thrice weekly, BEZ235 (40 mg/kg), BGT226 (10 mg/kg) and vehicle were administered daily. Six to 8 week old female NOD/SCID mice received 3 Gy of total body irradiation from an X-ray source delivered by a self contained cabinet (model X-RAY 320, Precision X-ray Inc, CMS Alphatech Pty Ltd, Sydney, Australia), equipped with a Pantak Seifert ISOVOLT 320 HS x-ray tube, 24 h before administration of 3-5×10^6^ human leukemic cells via tail vein injection. Mice were bled weekly and the percentage of human cells determined by flow cytometry using antibodies to human CD19 and murine CD45.

In survival assays, treatment commenced once ≥1% leukemic cells were detected in the peripheral blood (PB). Groups of 6 mice received vehicle only, BEZ235, BGT226 or everolimus until sacrifice was required due to deterioration of health scores. Mouse welfare was assessed daily using standardized score sheets for signs of leukemia including paralysis, loss of weight, ruffled coat, hunched posture, altered respiration and inactivity.

In functional assays, treatment was delayed until the mice had an expected survival time of less than 2 weeks. Mice were sacrificed 2 hours or 1 day after starting treatment. PB and spleens were analyzed for the presence of leukemic cells by flow cytometry. Spleen cells were collected for flow cytometry and Western blot analysis. Vertebral bodies and sternums were analyzed for ultra-structural changes affecting leukemic cells by light microscopy and transmission electron microscopy (TEM). Livers and femurs were collected for histological examination.

### Electron Microscopy

Electron microscopy was performed as previously described [10]. Briefly, cells were fixed in modified Karnofsky fixative (2.5% formaldehyde prepared freshly from paraformaldehyde, 2% EM grade glutaraldehyde in 0.1 M 3-[N-morpholino] propane sulphonic acid buffer, pH 7.4) for 2 hours. Cell blocks were post-fixed in osmium tetroxide, dehydrated in increasing concentrations of ethanol, and embedded in epoxy resin. Semi-thin (500 nm) sections were cut on a Reichert ultracut microtome and assessed by light microscopy. Ultrathin (80-90 nm) sections were cut and grid stained with 2% ethanolic uranyl acetate and then Reynolds lead citrate. The ultrastructure was examined using a Philips CM-10 transmission electron microscope (FEI, Portland OR) operated at 80 kV. Images were recorded with a Megaview G2 CCD camera (Olympus-SIS, Münster Germany).

### Statistical Analysis

Comparisons between 2 groups were performed using Student's t-tests and between multiple groups using ANOVA analysis. Where appropriate log transformation was made prior to analysis to stabilize to variance. Pairwise comparisons between groups were adjusted for multiple comparisons using Bonferroni's method. Survival was measured from the onset of disease until death and analyzed using SPSS, Version 15.0. The Kaplan-Meier method was used to construct survival curves, and results were compared using the log-rank test of survival distribution by treatment stratified by xenograft. The number of cells containing acidic vacuoles was compared between groups using Student T tests and comparison of the number of acidic vacuoles/cell analyzed using the Kruskal-Wallis test, to determine differences between treatments, and the Jonckheere-Terpstra test to demonstrate association between increasing numbers of acidic vacuoles/cell with drug treatment.

## SUPPLEMENTARY MATERIAL, TABLE AND FIGURES


