# Supplementary material for: Efficacy of dual PI-3K and mTOR inhibitors in vitro and in vivo in acute lymphoblastic leukemia
Source: Oncotarget. 2014 Jul 25;5(21):10460–72. doi: 10.18632/oncotarget.2260 (PMC4279386; doi:10.18632/oncotarget.2260)
Supplement: Supplementary file 1 [file oncotarget-05-10460-s001.pdf]

Efficacy of dual PI-3K and mTOR inhibitors *in Vitro* and *in Vivo* in acute lymphoblastic leukemia

Supplementary Material

Table S1: Clinical information

Patient ID	Sex/Age	Immuno-phenotype	Cytogenetics
**1786**	F/12	$CD10^+CD34^-$ $CD20^+$	No metaphases
**2070**	M/65	$CD34^+CD10^+$ $CD20^+$ $CD45^+$	45XY t(9;22)(q34;q11.2)del(9)(p21)
**0398**	M/15	$CD10^-CD34^+$	46XY add(3)(q29)t(14:19)(q32p13)
**1196**	F/8	$CD10^+CD34^-$	46XX -19,del(19),t(1;19)(q23p13)
**1345**	F/5	$CD10^+$	45XX dup(1)(q42q25),del(3)(q21),-9,del(9)(p22), t(18;20)(q21q13.1)
**1809**	M/12	$CD10^+CD34^+$	46XY,del(4)(q21q25),-9,add(13)(q14),+add(22)(p13)[9] 46XY[11]
**0407**	M/45	$CD10^+CD34^-$	t(1;19)[1]
**2032**	M/12	$CD10^+CD34^-$	46XYadd(9)(p24),del(9)(p21),del(13)(q11q21),der(19) t(1;19)(q23;p13)

[1]Obtained by PCR.

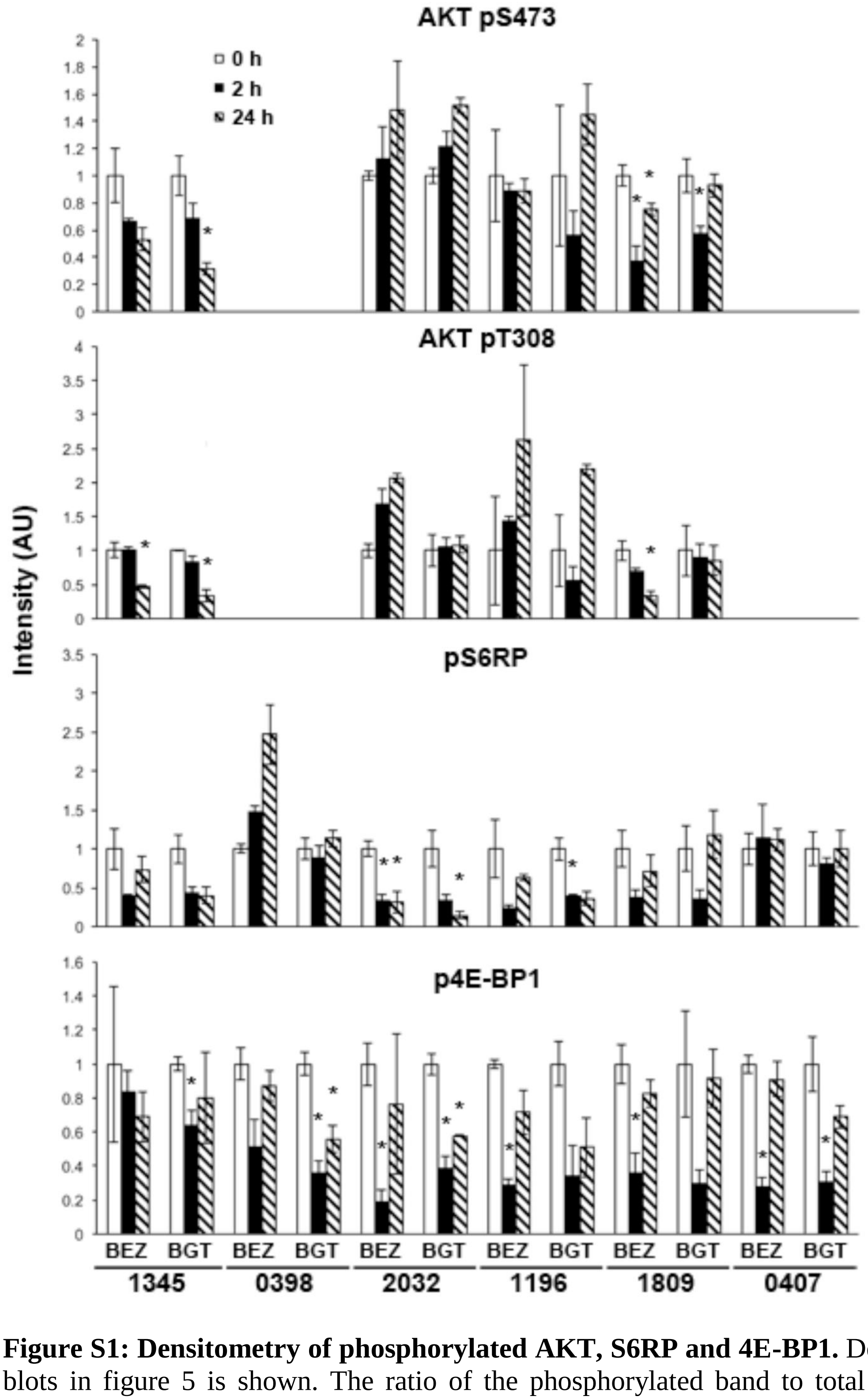

Figure S1: Densitometry of phosphorylated AKT, S6RP and 4E-BP1. Densitometry of the blots in figure 5 is shown. The ratio of the phosphorylated band to total protein has been corrected for loading using GAPDH or β-actin as indicated in Figure 5. The mean ± SD of replicates is shown. *p<0.05 compared to control treated cells.

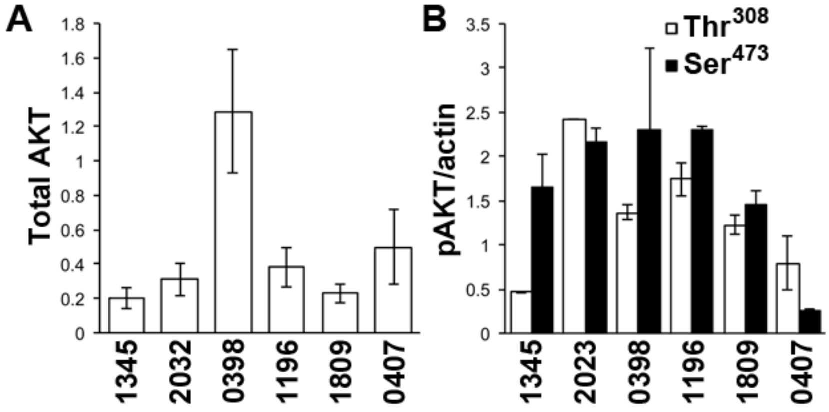

Figure S2: Densitometry of the blots in figure 6A is shown. (A) The ratio of the total AKT protein (A) or AKT phosphorylated of Thr^{308} or Ser^{473} (B) has been corrected for loading using beta-actin. The mean ± SD of replicates from two independent gels is shown.

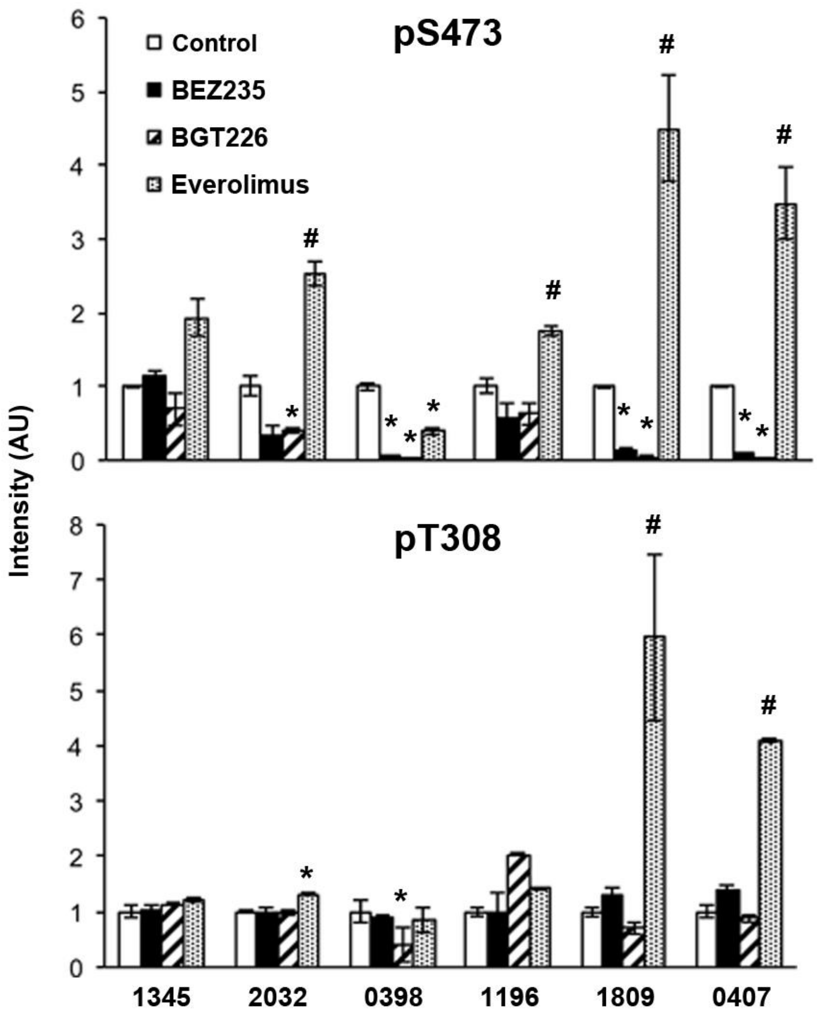

Figure S3: Densitometry of the blots in figure 6 is shown. The ratio of the phosphorylated band to total protein has been corrected for loading using GAPDH or beta-actin as indicated in Figure 6. The mean ± SD of replicates is shown. *p<0.05 compared to control treated cells.
